# Supplementary material for: NMR and MS reveal characteristic metabolome atlas and optimize esophageal squamous cell carcinoma early detection
Source: Nat Commun. 2024 Mar 19;15:2463. doi: 10.1038/s41467-024-46837-0 (PMC10951220; doi:10.1038/s41467-024-46837-0)
Supplement: Supplementary file 1 — Supplementary Information [file 41467_2024_46837_MOESM1_ESM.pdf]

**NMR and MS reveal characteristic metabolome atlas and optimize esophageal squamous cell carcinoma early detection**

Yan Zhao[#1,2], Changchun Ma[#3], Rongzhi Cai[1], Lijing Xin[4], Yongsheng Li[5], Lixin Ke[1], Wei Ye[1], Ting Ouyang[1], Jiahao Liang[1], Renhua Wu[*1], Yan Lin[*1]

1. Radiology Department, Second Affiliated Hospital of Shantou University Medical College, Shantou, Guangdong, China
2. Central Laboratory, Clinical Research Center, Shantou Central Hospital, Shantou, Guangdong, China
3. Radiation Oncology Department, Cancer Hospital of Shantou University Medical College, Shantou, Guangdong, China
4. Animal Imaging and Technology Core, Center for Biomedical Imaging, Ecole Polytechnique Fédérale de Lausanne, Lausanne, Switzerland.
5. Department of Medical Oncology, Chongqing University Cancer Hospital, Chongqing, China

**Supplementary information**

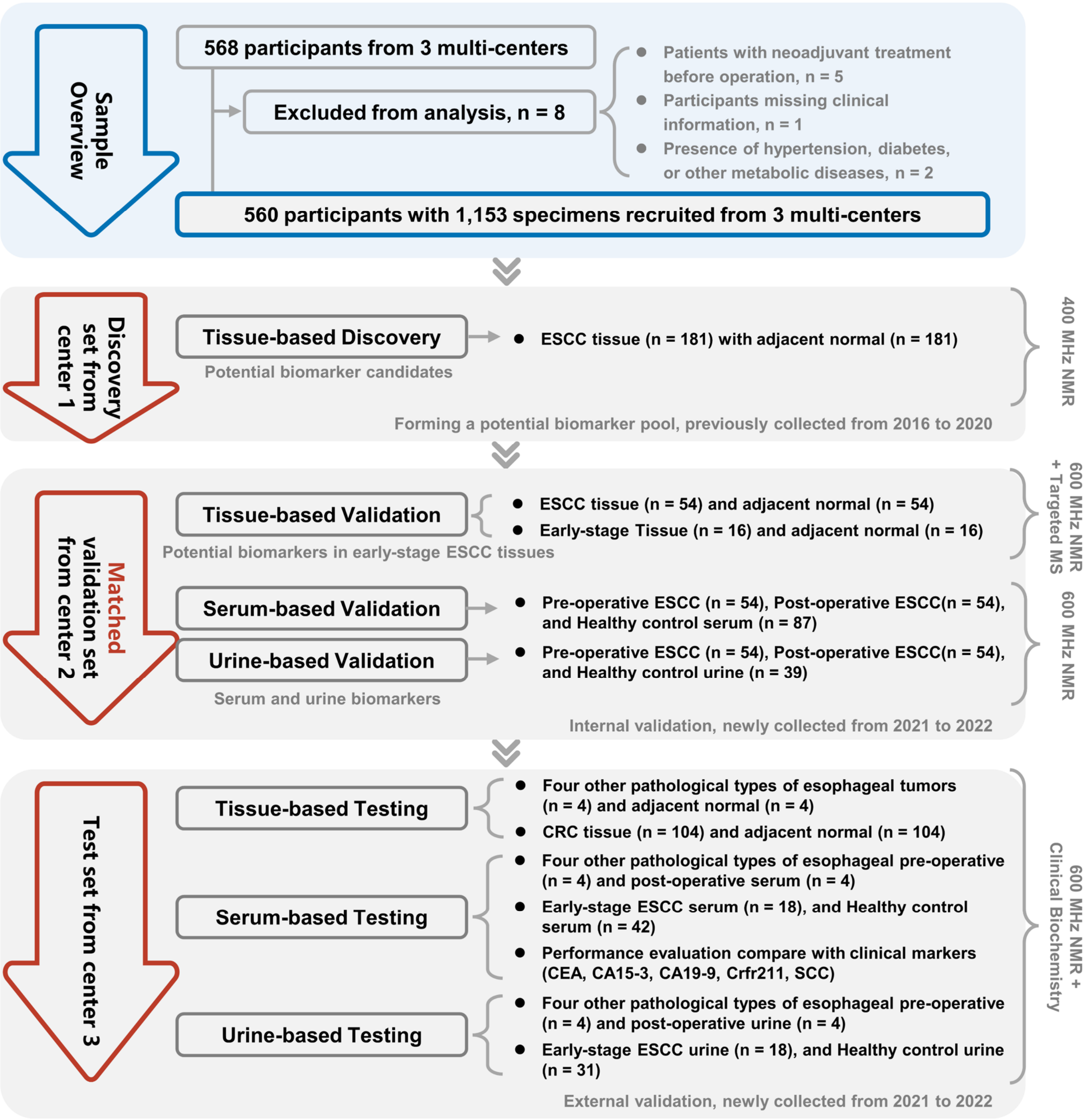


**Supplementary Figure 1**. Flowchart and sample distribution in the discovery, validation and test sets for developing biofluid classifiers for ESCC early detection. A total of 560 participants with 1153 specimens recruited from 3 multi-centers were in the discovery, validation and test sets.

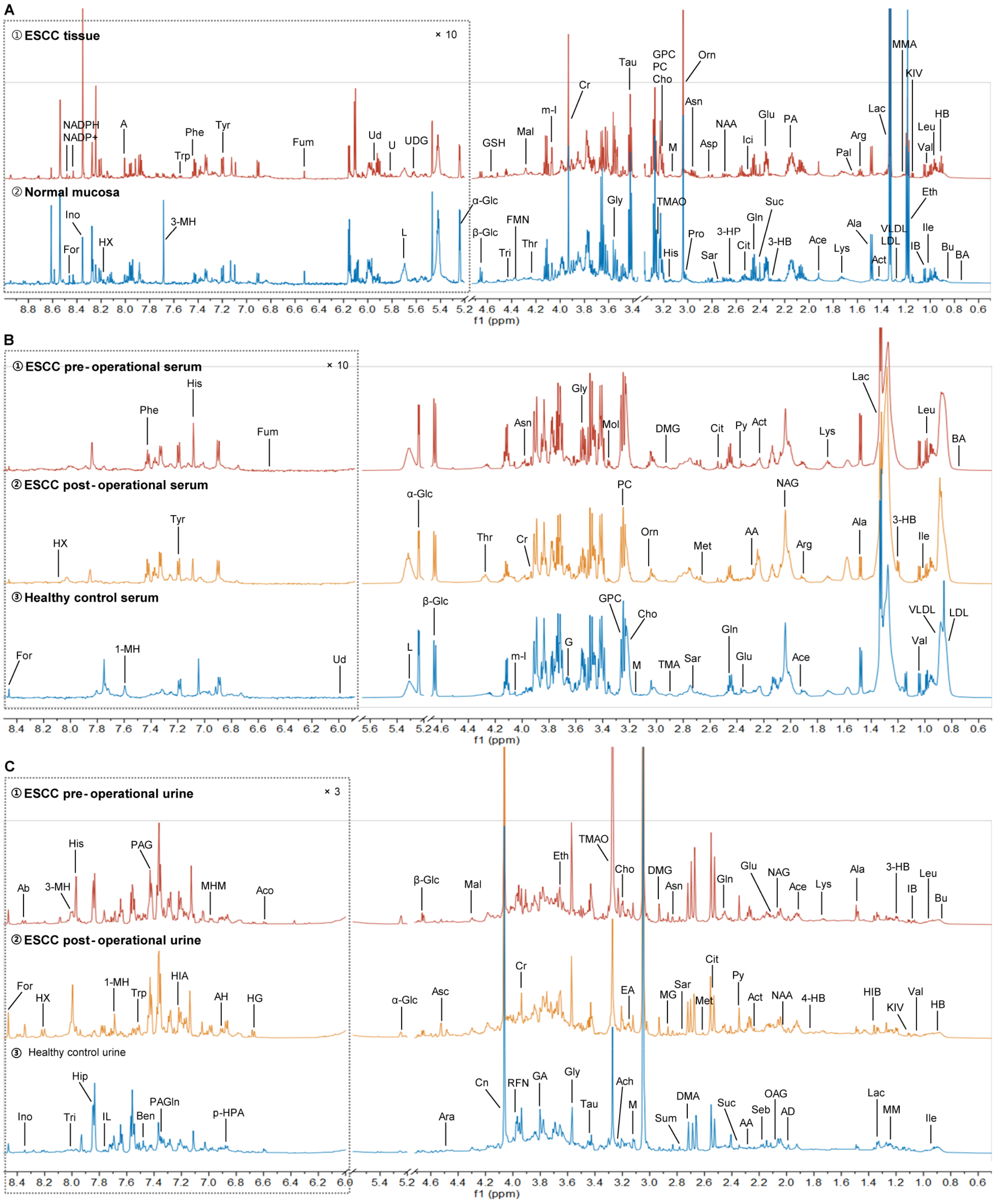


**Supplementary Figure 2**. Representative 600 MHz 1D $^{1}$H NMR spectra (δ 0.5–9.0) of tissue (A), serum (B) and urine (C) obtained from ESCC patients and healthy controls. The metabolites were assigned, and the characteristic peaks were labelled.

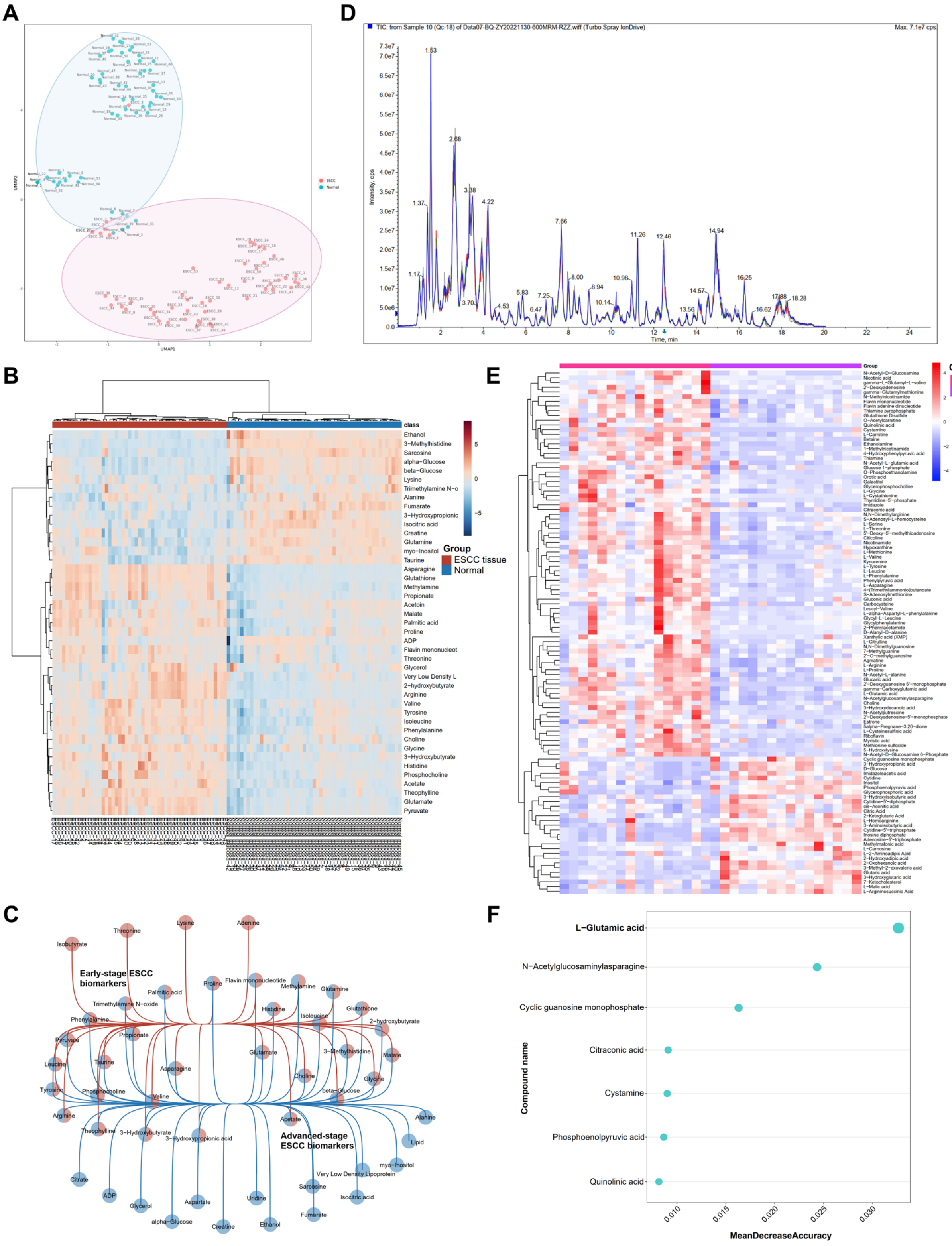


**Supplementary Figure 3**. Quality control and machine learning-based classification analysis in NMR- and MS-based metabolomic profiles.

**(A)** UMAP dimensional reduction plot utilizing measured metabolites in the validation set, derived from $^{1}$H-NMR spectra of ESCC tissue extracts. Red: ESCC; Blue: HC.

**(B)** Unsupervised hierarchical clustering of ESCC and normal mucosa groups across NMR-based metabolite integration (Ward's method clustering). Red: ESCC tissue; Blue: Normal tissue.

**(C)** Independent or overlapping potential biomarkers between early and late ESCCs. Red: Early-stage; Blue: Advanced-stage.

**(D)** The total ion chromatogram (TIC) plot of QC samples from the MS-based metabolic profile displaying the retention time on the x-axis and peak intensity on the y-axis.

**(E)** Heatmap presents targeted MS-based metabolite concentrations between early stage ESCC and normal mucosa. Pink: Early tumor; Purple: Normal.

**(F)** RF classifier identifies critical metabolites in the targeted metabolome for distinguishing early stage ESCC from normal tissue. A higher mean decrease accuracy (MDA) value indicates a greater significance of the variable. Source data are provided as a Source Data file.

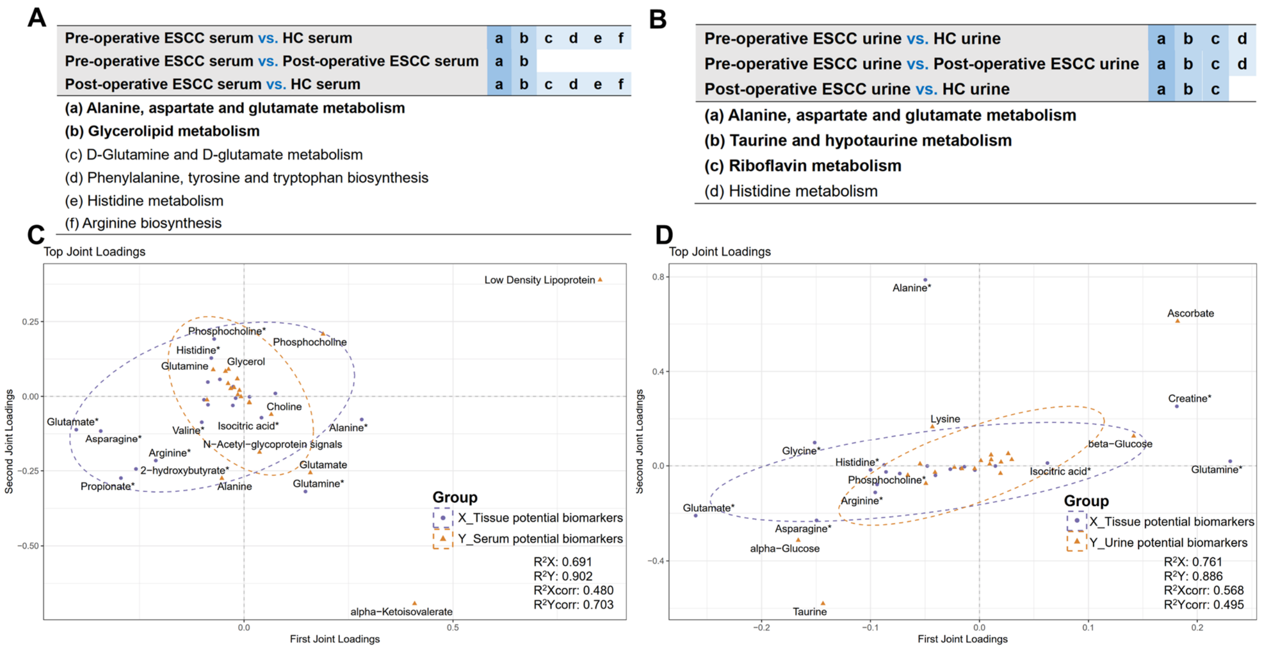


**Supplementary Figure 4**. Perturbed metabolic pathways in serum and urine from tumor-bearing states to post-operation and to healthy states.

**(A-B)** Disturbed metabolic pathways of differential serum (A) or urine (B) metabolites were associated with variations in physiological states.

**(C-D)** O2PLS analyses across tissue and serum or urine. The top 30 metabolites were labelled. Purple: potential biomarkers confirmed in early-ESCC tissue; orange: metabolites detected in early-ESCC serum (C) or urine (D) by NMR. Source data are provided as a Source Data file.

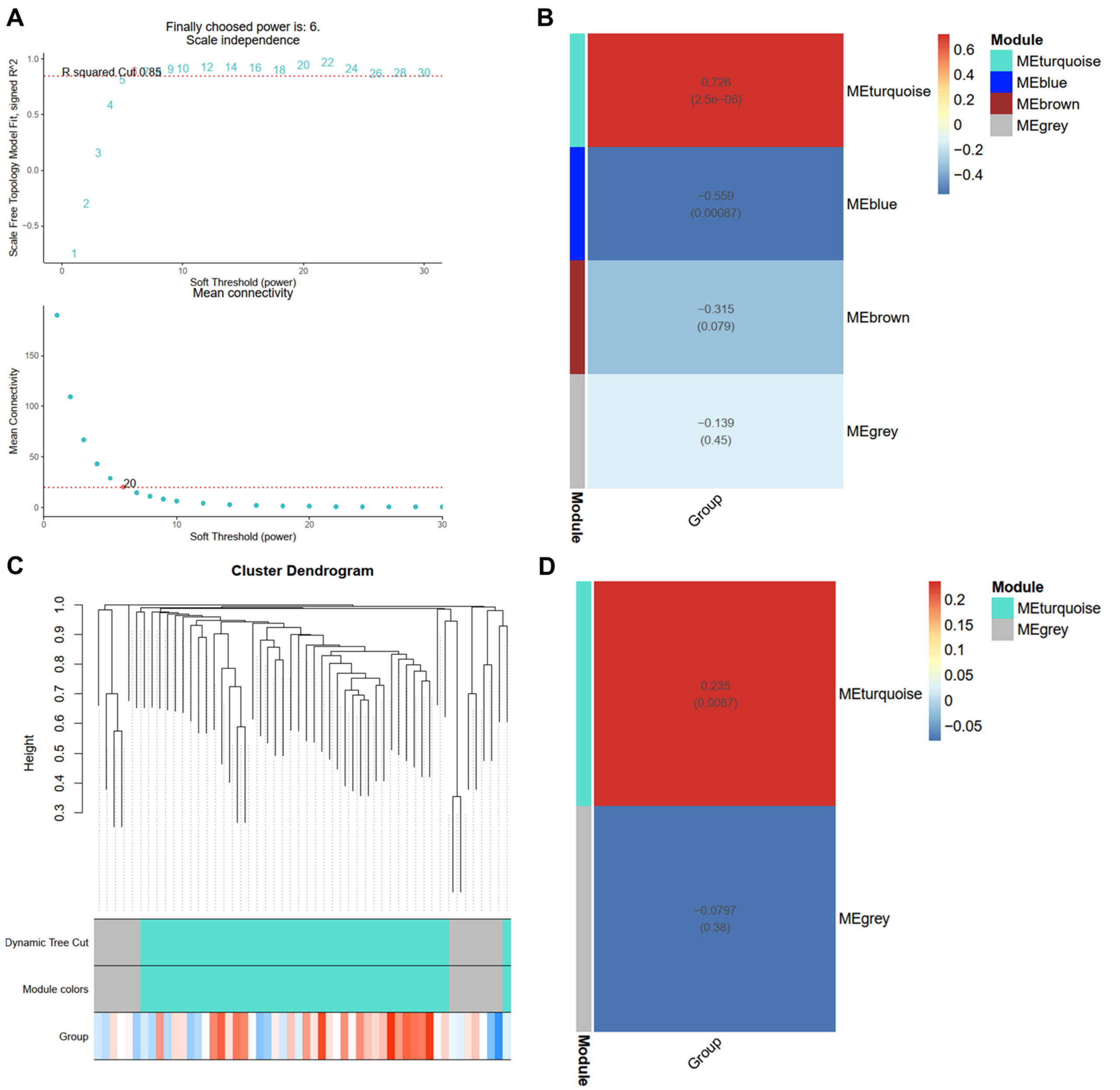


**Supplementary Figure 5**. Supplementary results of WGCNA characterizing metabolic modules.

**(A)** Early ESCC tissue vs. normal mucosa: scale independence and mean connectivity calculated for samples during WGCNA analysis. A threshold power of $\beta = 6$ was used for generating the co-expression networks.

**(B)** Early ESCC tissue vs. normal mucosa: module - trait association. Each row corresponds to a colored module eigen-metabolite. The column represents grouping. The correlation coefficients between the module and trait are shown in red for positive correlations and blue for negative correlations. The numbers within each colored box give the *p*-values for the statistical significance of each correlation.

**(C)** Pre-operative vs. post-operative serum: WGCNA cluster dendrogram groups differential metabolites into distinct metabolite modules defined by dendrogram branch cutting. Turquoise was the only strongly associated module between the pre- and post-operative serum group.

**(D)** Pre-operative vs. post-operative serum: module - phenotype association. Source data are provided as a Source Data file.

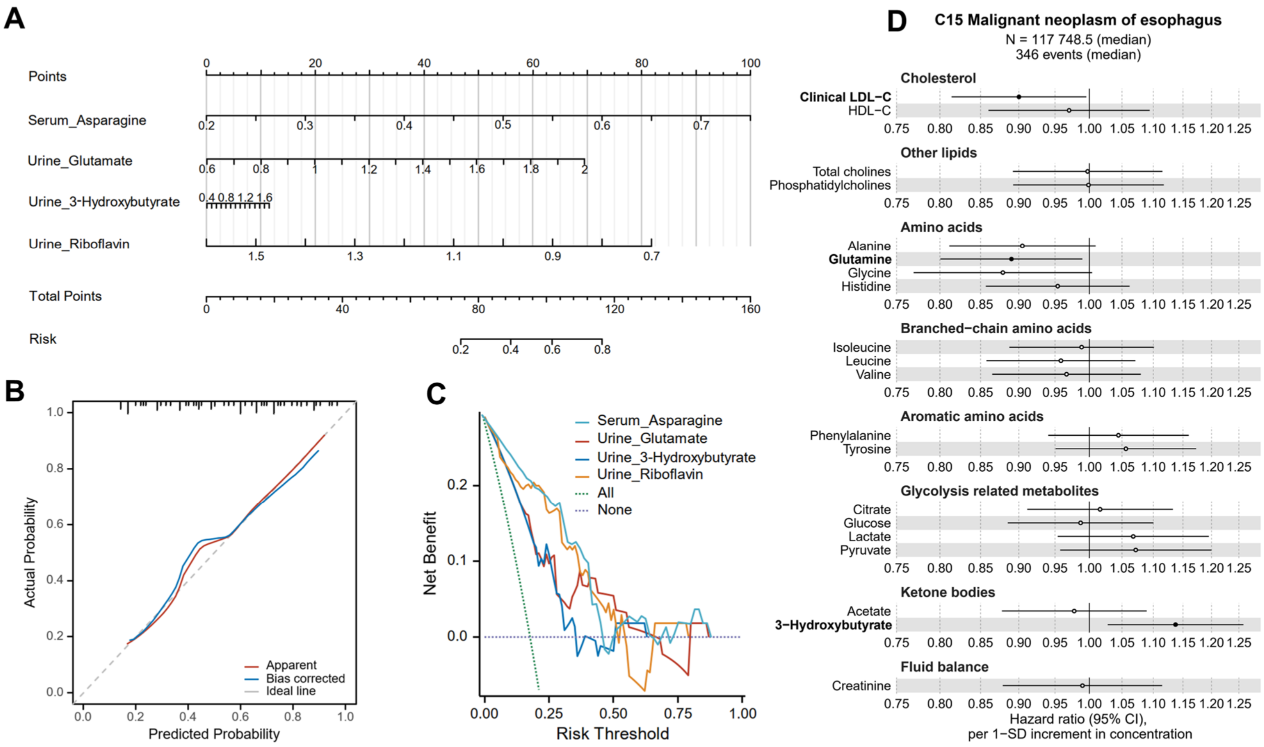


**Supplementary Figure 6**. UK Biobank prospective data support the protective and risk factors identified in this study.

**(A)** The metabolic nomogram was developed with the serum (asparagine) and urine (glutamate, 3-hydroxybutyrate, riboflavin) signatures to predict the risk of early stage ESCC. The probability of early-stage ESCC for a given patient was estimated by locating the integration of metabolites and drawing a line straight up to the Points axis to determine the associated score with that number; the scores were then summed and this sum was located on the Total Points axis. Subsequently, a vertical line was drawn down to the Risk axis, and the probability was read off.

**(B)** Calibration curves of the metabolic nomogram model for predicting ESCC.

**(C)** DCA plot demonstrating the clinical utility of the predictive model for ESCC. The x-axis represents the risk threshold probability, while the y-axis corresponds to the net benefit. The solid line represents the decision curve for the biofluid features, while the dashed lines represent the two reference strategies: 'diagnose all' and 'diagnose none'.

**(D)** Metabolites detected by NMR associations with future malignant neoplasm of esophageal events. Hazard ratios of 20 metabolites measured with incident esophageal cancer in the UK Biobank study (n = 117,748.5, 346 events). Hazard ratios were per 1-SD metabolite concentration. Error bars denote 95% CI. Metabolites indicated in bold font: $p < 0.05$ (multiple testing correction). LDL, low-density lipoprotein; SD, standard deviation. Source data are provided as a Source Data file.

**Supplementary Table 1**. The demographics and baseline characteristics of the discovery, validation and test sets.

| Variables | Discovery set from center 1 | Matched validation set from center 2 | | Test set from center 3 | | | HCs |
|---|---|---|---|---|---|---|---|
| Statistics – No. | | | | | | | |
| Participants (n = 560) | 181 ESCC patients | 54 ESCC patients | | 104 CRC patients | 4 other pathological EC* patients | 18 early ESCC patients | 199 healthy volunteers |
| Specimens (n = 1153) | 362 ESCC tissue samples | 324 matched tissue, pre- and post-operative serum and urine samples | | 208 CRC tissue samples | 24 matched tissue, pre- and post-operative serum and urine samples | 18 early ESCC serum samples,<br>18 early ESCC urine samples | 87+42 HC serum samples,<br>39+31 HC urine samples |
| Gender – No. (%) | | | | | | | |
| Male | 109 (60.22%) | 12 (75.00%) | 32 (84.21%) | 49 (47.12%) | 3 (75.00%) | 12 (66.67%) | 115 (57.79%) |
| Female | 72 (39.78%) | 4 (25.00%) | 6 (15.79%) | 55 (52.88%) | 1 (25.00%) | 6 (33.33%) | 84 (42.21%) |
| Age | | | | | | | |
| Median (IQR) | 61 (51-71) | 65 (58-69.5) | 67 (57-71) | 62 (52-72.5) | 69 (65-75.5) | 68 (62.5-70) | 60 (49.5-71) |
| BMI, kg/m$^2$ | | | | | | | |
| Mean ± SD | 27.2 ± 4.5 | 20.4 ± 1.9 | 22.1 ± 2.6 | 25.3 ± 3.1 | 22.30 ± 3.1 | 21.9 ± 2.3 | 21.6 ± 2.0 |
| Tumor stage – No. (%) | | | | | | | |
| Stage I/II | 108 (59.67%) | 16 | | 32 (30.77%) | | 18 | |
| Stage III/IV | 73 (40.33%) | | 38 | 72 (69.23%) | | | |
| Tumor topographical site – No. (%) | | | | | | | |
| Cervical | 6 (3.31%) | 0 (0.00%) | 0 (0.00%) | | | 0 (0.00%) | |
| Upper thoracic | 26 (14.36%) | 3 (18.75%) | 3 (7.89%) | | | 3 (16.67%) | |
| Middle thoracic | 95 (52.49%) | 11 (68.75%) | 23 (60.53%) | | | 11 (61.11%) | |
| Lower thoracic | 54 (29.84%) | 2 (12.50%) | 12 (31.58%) | | | 4 (22.22%) | |
| Clinical blood markers – Positive No. (%) | | | | | | | |
| CEA (0-3.8 ng/mL; Smoker 0.0-5.5 ng/mL) | 60 (33.15%) | 1 (6.25%) | 3 (7.89%) | 31 (29.80%) | 0 (0.00%) | 1 (5.56%) | |
| CA 19-9 (0-27 U/mL) | 34 (18.78%) | 0 (0.00%) | 2 (5.26%) | 12 (11.54%) | 0 (0.00%) | 0 (0.00%) | |
| CA15-3 (0-25 U/mL) | | 0 (0.00%) | 1 (2.26%) | | 0 (0.00%) | 0 (0.00%) | |
| SCC (0.5-2.7 ng/mL) | | 0 (0.00%) | 0 (0.00%) | | | 0 (0.00%) | |
| CYFRA21-1 (0-3.3 ng/mL) | | 3 (18.75%) | 9 (23.68%) | | 1 (25.00%) | 2 (11.11%) | |
| PD-L1 (CPS≥10, may benefit from PD-1/PD-L1 inhibitor) | | 0 (0.00%) | 4 (10.53%) | | | 0 (0.00%) | |

Abbreviations: ESCC: Esophageal squamous cell carcinoma; CRC: Colorectal cancer; HC: Healthy Control; BMI: Body mass index; CEA: Carcinoembryonic antigen; CA 19-9: Carbohydrate antigen 19-9; CA 15-3: Carbohydrate antigen 15-3; CYFRA21-1: Cytokeratin-19-fragment CYFRA21-1; SCC: Squamous cell carcinoma *antigen;* CPS: Combined positive score; IQR: Interquartile range; SD: Standard deviation.

Note: * including Esophageal adenocarcinoma (EAC), Adenocarcinoma of the esophagogastric junction (GEJ), undifferentiated carcinoma of esophagus, esophageal stromal tumors, and the corresponding normal mucosa.

**Supplementary Table 2**. Clinical phenotypes of the matched validation set.

| Variables | Matched ESCC samples in the validation set | |
|---|---|---|
| | Early (n = 16) | Advanced (n = 38) |
| Family history of ESCC, Positive No. (%) | 0 (0.00%) | 2 (0.053%) |
| Smoke - No. (%) | 11 (68.75%) | 26 (68.42%) |
| Alcohol - No. (%) | 8 (50.00%) | 17 (44.74%) |
| Area, No. (%) | | |
| Urban areas | 3 (18.75%) | 8 (21.05%) |
| Rural areas | 13 (81.25%) | 30 (78.95%) |
| Clinical symptoms, No. (%) | | |
| Dysphagia | 16 (100%) | 35 (92.10%) |
| Odynophagia | 2 (12.50%) | 1 (2.63%) |
| Vomiting | 3 (18.75%) | 2 (5.26%) |
| Weight loss | 8 (50.00%) | 13 (34.21) |
| Time from symptom appearance to admission | | |
| Within 1 month | 1 (6.25%) | 5 (13.16%) |
| 1-6 months | 13 (81.25%) | 27 (71.05%) |
| Over 6 months | 2 (12.50%) | 6 (15.79%) |
| Histologic grade | | |
| G1 (Grade 1, well differentiated) | 4 (25.00%) | 5 (13.15%) |
| G2 (Grade 2, moderately differentiated) | 9 (56.25%) | 23 (60.53%) |
| G3 (Grade 3, poorly differentiated) | 3 (18.75%) | 10 (26.32%) |
| Radiological signs before surgery, Median (cm, IQR) | | |
| Thickest segment of the esophageal wall | 1.0 (0.80-1.50) | 1.45 (1.00-1.85) |
| Esophageal stenosis/lesion length | 4.0 (3.00-6.00) | 5.85 (5.00-8.13) |
| Maximum diameter of each lymph node | 0.7 (0.40-1.00) | 1.15 (0.78-1.50) |
| Pre-operative endoscopic biopsy showed only IN, No. (%) | 2 (12.5%) | 4 (10.53%) |
| Thoracotomy | | |
| Yes | 0 (0.00%) | 5 (13.16) |
| No | 16 (100%) | 33 (86.84%) |
| Transfer to ICU post-operative | | |
| Yes | 0 (0.00%) | 11 (28.95%) |
| No | 16 (100%) | 27 (71.05%) |

Abbreviations: ESCC: Esophageal squamous cell carcinoma; IN: Intraepithelial neoplasia; ICU: Intensive Care Unit; IQR: Interquartile range.

**Supplementary Table 3**. Comparison of the theoretically low-performing simplified biofluid panels using different machine learning methods.

| Model Metrics | Efficacy validation of serum panel | | | | Efficacy validation of urine panel | | | |
|---|---|---|---|---|---|---|---|---|
| | Logistic regression | XGboost | Gaussian Naive Bayes | K-Nearest Neighbors | Logistic regression | XGboost | Gaussian Naive Bayes | K-Nearest Neighbors |
| AUC (95% CI) | 0.984 (0.968 - 1.000) | 0.979 (0.905-1.000) | 0.977 (0.892-1.000) | 0.944 (0.867-1.000) | 0.930 (0.913 - 0.976) | 0.842 (0.715-1.000) | 0.874 (0.840-0.908) | 0.826 (0.789-0.963) |
| SE | 0.955 | 0.947 | 0.948 | 0.931 | 0.850 | 0.848 | 0.831 | 0.729 |
| SP | 0.943 | 0.939 | 0.929 | 0.819 | 0.949 | 0.894 | 0.863 | 0.933 |
| Accuracy | 0.948 | 0.929 | 0.922 | 0.907 | 0.889 | 0.932 | 0.848 | 0.841 |
| PPV | 0.926 | 0.893 | 0.889 | 0.965 | 0.962 | 0.923 | 0.846 | 0.706 |
| NPV | 0.965 | 0.948 | 0.948 | 0.919 | 0.804 | 0.747 | 0.833 | 0.725 |

Abbreviations: AUC: Areas Under the Curve; SE: Sensitivity; SP: Specificity; PPV: Positive predictive value; NPV: Negative predictive value

**Supplementary Table 4**. Prediction probability values and outcomes of the blinded cases by the biofluid metabolic classifiers.

| Blinded case | Probability scores | Predicted class |
|---|---|---|
| Serum – 1 | 0.85158 | Early_ESCC |
| Serum – 2 | 0.95892 | Early_ESCC |
| Serum – 3 | 0.95902 | Early_ESCC |
| Serum – 4 | 0.89986 | Early_ESCC |
| Serum – 5 | 0.95378 | Early_ESCC |
| Serum – 6 | 0.97922 | Early_ESCC |
| Urine – 1 | 0.57677 | Early_ESCC |
| Urine – 2 | 0.90475 | Early_ESCC |
| Urine – 3 | 0.9571 | Early_ESCC |
| Urine – 4 | 0.79 | Early_ESCC |
| Urine – 5 | 0.81501 | Healthy control (prediction error) |
| Urine – 6 | 0.87018 | Early_ESCC |
