# Supplementary material for: NMR and MS reveal characteristic metabolome atlas and optimize esophageal squamous cell carcinoma early detection
Source: Nat Commun. 2024 Mar 19;15:2463. doi: 10.1038/s41467-024-46837-0 (PMC10951220; doi:10.1038/s41467-024-46837-0)
Supplement: Supplementary file 3 — Reporting Summary [file 41467_2024_46837_MOESM3_ESM.pdf]

nature portfolio

Corresponding author(s): Yan Lin, Renhua Wu

Last updated by author(s): Feb 13, 2024

Reporting Summary

Nature Portfolio wishes to improve the reproducibility of the work that we publish. This form provides structure for consistency and transparency in reporting. For further information on Nature Portfolio policies, see our Editorial Policies and the Editorial Policy Checklist.

Statistics

For all statistical analyses, confirm that the following items are present in the figure legend, table legend, main text, or Methods section.

n/a	Confirmed	
☐	☒	The exact sample size (*n*) for each experimental group/condition, given as a discrete number and unit of measurement
☐	☒	A statement on whether measurements were taken from distinct samples or whether the same sample was measured repeatedly
☐	☒	The statistical test(s) used AND whether they are one- or two-sided<br>*Only common tests should be described solely by name; describe more complex techniques in the Methods section.*
☐	☒	A description of all covariates tested
☐	☒	A description of any assumptions or corrections, such as tests of normality and adjustment for multiple comparisons
☐	☒	A full description of the statistical parameters including central tendency (e.g. means) or other basic estimates (e.g. regression coefficient) AND variation (e.g. standard deviation) or associated estimates of uncertainty (e.g. confidence intervals)
☐	☒	For null hypothesis testing, the test statistic (e.g. *F*, *t*, *r*) with confidence intervals, effect sizes, degrees of freedom and *P* value noted<br>*Give P values as exact values whenever suitable.*
☒	☐	For Bayesian analysis, information on the choice of priors and Markov chain Monte Carlo settings
☒	☐	For hierarchical and complex designs, identification of the appropriate level for tests and full reporting of outcomes
☐	☒	Estimates of effect sizes (e.g. Cohen's *d*, Pearson's *r*), indicating how they were calculated

Our web collection on statistics for biologists contains articles on many of the points above.

Software and code

Policy information about availability of computer code

Data collection	Bruker Advance NMR spectrometer (Bruker BioSpin, Germany) equipped with a triple resonance cryogenic probe operating at 600.13 MHz and 298.0 K was used for 1H-NMR spectra data collection. The NMR data acquisition was performed using TopSpin 3.2 software.<br>For LC-MS Analysis, metabolic extracts were analyzed using an ACQUITY UPLC H-Class (Waters) ultra-high-performance liquid chromatography system, utilizing the Atlantis Premier BEH Z-HILIC Column (Waters, 1.7 µm, 2.1 mm × 150 mm) for chromatographic separation of target compounds. AB SCIEX 6500 QTRAP+ triple quadrupole MS equipped with IonDrive Turbo V ESI ion source was used for MS analysis in MRM mode. For GC-MS Analysis, metabolic extracts were analyzed using the SHIMADZU GC2030-QP2020 NX gas chromatography-mass spectrometer. All MS data collection and quantitative analysis of target compounds were performed using SCIEX Analyst Work Station Software (version 1.7.2) and BIOTREE Bio Bud (version 2.1.4).
Data analysis	The NMR spectra were processed using MestReNova (version 14.0, Mestrelab Research, Spain) and subsequently analyzed in SIMCA (version 14.1, Umetrics, Sweden) for pattern recognition analyses. The quantitative analysis of targeted compounds was performed using BIOTREE Bio Bud (2.1.4). Univariate statistical analyses were performed using SPSS software (version 26, IBM SPSS Statistics, USA). Biomarker analysis and KEGG pathway analysis were analyzed by MetaboAnalyst 6.0 (https://www.metaboanalyst.ca). ROC analysis was performed by pROC (v.1.18.0.) R package. A multivariable logistic regression analysis was performed using the glm function. Nomogram was developed based on the logistic model, utilizing the rms R package for visualization. O2PLS was performed by OmicsPLS R package. Mantel test was performed on https://www.genescloud.cn/chart/NetHeatmap. WGCNA was performed by WGCNA R package. KEGG pathway was plotted using the OmicStudio tools at https://www.omicstudio.cn/tool.

For manuscripts utilizing custom algorithms or software that are central to the research but not yet described in published literature, software must be made available to editors and reviewers. We strongly encourage code deposition in a community repository (e.g. GitHub). See the Nature Portfolio guidelines for submitting code & software for further information.

Data

Policy information about availability of data

All manuscripts must include a data availability statement. This statement should provide the following information, where applicable:

- Accession codes, unique identifiers, or web links for publicly available datasets
- A description of any restrictions on data availability
- For clinical datasets or third party data, please ensure that the statement adheres to our policy

The matched NMR and MS metabolomics data generated in this study have been deposited in the NIH Common Fund's National Metabolomics Data Repository (NMDR) website, the Metabolomics Workbench database under accession code Project ID PR001876: http://dx.doi.org/10.21228/M87426. The NMR data from the previous study and the smaller subset of the test set are not available due to intellectual property agreements with different hospitals. Still, they can be obtained from the corresponding author upon reasonable request. The processed metabolomics data are provided in the Supplementary Information/Source Data file. The RNA-seq data from TCGA-ESCA and GTEx databases can be downloaded from https://xenabrowser.net/datapages/.

Research involving human participants, their data, or biological material

Policy information about studies with human participants or human data. See also policy information about sex, gender (identity/presentation), and sexual orientation and race, ethnicity and racism.

Reporting on sex and gender	Gender information was collected based on the informed consent of all participants, who were matched as closely as possible in age and gender and were presented in Supplementary Table 1. However, as our study aimed to establish a classifier to detect early-stage ESCC in a general population, gender/sex was not included as a parameter for model training. The model also achieved a desirable AUC in both internal and external validation sets, indicating the model's applicability to a general population.
Reporting on race, ethnicity, or other socially relevant groupings	In this study, we included 560 participants from three centers in southern China. The population information was collected through self-reporting and patients' electronic medical records.
Population characteristics	We studied 1,153 samples from 560 participants across three medical centers in southern China. The distribution of data was as follows. Discovery set from center 1 (n = 362 biologically independent samples), Validation set from center 2 (n = 450 biologically independent samples), and Test set from center 3 (n = 341 biologically independent samples). Detailed characteristics are provided in Supplementary Table 1, 2.
Recruitment	Discovery set from center 1 was collected between 2016 and 2020. Validation set from center 2 was obtained between 2021 and 2022. Test set from center 3 were collected between 2021 and 2022.<br>The diagnosis of patients was made using endoscopy combined with biopsy, imaging, clinical symptoms, medical history, and further confirmed by histopathological examination of the surgically resected specimen. Healthy subjects from the high-incidence coastal area of ESCC were also included, and all clinical examinations were within normal ranges. The exclusion criteria were as follows: (1) subjects with a current or past history of other malignancies and history of gastrointestinal operations, (2) patients with neoadjuvant treatment before operation, (3) participants missing clinical information, (4) bacterial infection or use of antibiotics or probiotics within one month before surgery, and (5) presence of hypertension, diabetes, or other metabolic diseases. These exclusion criteria were consistent across all three datasets, and the study participants were consecutively and randomly enrolled, with well-matched age and gender as closely as possible.<br>We rigorously applied inclusion and exclusion criteria to ensure that the study population is closely representative of the target population, thereby minimizing selection bias. In addition, this project implemented standardized sample collection, processing procedures, and rigorous quality control in data analysis to mitigate information bias. Informed consents were obtained from all participants and no compensation was provided.
Ethics oversight	The study protocol has been approved by the Ethics Committee of Shantou University Medical College (#2021-92, #2022-103) and registered at the Chinese Clinical Trial Registry (Registration number: ChiCTR2300073613). Sample collection followed established biobanking protocols and ethical and legal standards, following informed written consent. The report follows the Standards for Reporting of Diagnostic Accuracy Studies (STARD) reporting guideline.

Note that full information on the approval of the study protocol must also be provided in the manuscript.

Field-specific reporting

Please select the one below that is the best fit for your research. If you are not sure, read the appropriate sections before making your selection.

☒ Life sciences ☐ Behavioural & social sciences ☐ Ecological, evolutionary & environmental sciences

For a reference copy of the document with all sections, see nature.com/documents/nr-reporting-summary-flat.pdf

Life sciences study design

All studies must disclose on these points even when the disclosure is negative.

Sample size	Regarding the sample size estimation, we used the PASS (Power Analysis and Sample Size) software and relevant formulas to estimate the

Sample size	sample size based on the standard deviation, discrimination, test level, and test efficiency of metabolic biomarkers detection obtained in our previous work. When the sample size estimation parameters were set to specificity of 90%±10%, sensitivity of 80%±10%, significance level (α) = 0.05, confidence level (1-α) of 0.95, and two-sided test type, the required sample size per group was estimated to be n = 44. We tried to make the sample size of multiple subgroups exceed this threshold in this project.<br>Moreover, due to the limited sample size of early-stage ESCC patients, metabolomics studies can be conducted with as few as 16 cases per group, according to a previous study in Nature Communications, which showed that considering sensitivity, reproducibility, detection limit, linearity and dynamic range, selectivity, identification, coverage, etc., the minimum optimal sample size for metabolomics and other omics studies was 16 cases, with an average power of at least 0.8, FDR of 0.05, and initial Cohen's d of 0.8.
Data exclusions	The preliminary inclusion for this study involved 568 participants from three centers, with eight individuals excluded for the following reasons: patients with neoadjuvant treatment before operation (n = 5), participants missing clinical information (n = 1), and presence of hypertension, diabetes, or other metabolic diseases (n = 2).
Replication	The number of biological replications are as indicated in figure legends and Methods with successful attempts. The reproducibility of tissue, serum and urine metabolomics data was based on the strict instrument parameters and quality control (See Methods and Supplementary Figure 3).<br>In this study, the 600 MHz NMR results aligned with our previous 400 MHz NMR results. Additionally, targeted MS analysis was conducted on the same batch of early-stage ESCC tissue from the validation set. The MS approaches confirmed the accuracy of the NMR results.
Randomization	The study participants were consecutively and randomly enrolled. Tissue samples were allocated to the NMR platform in random order in the discovery and validation sets. For paired analysis, the serum and urine samples from the same patient were subjected to corresponding NMR and targeted MS analyses, following the order of tissue samples.
Blinding	The histopathological results, which serve as clinical reference testing data, were kept confidential to individuals responsible for processing and setting up the metabolomics testing (index tests). The investigators were blinded to group allocation when performing NMR or MS data acquisition.

Reporting for specific materials, systems and methods

We require information from authors about some types of materials, experimental systems and methods used in many studies. Here, indicate whether each material, system or method listed is relevant to your study. If you are not sure if a list item applies to your research, read the appropriate section before selecting a response.

Materials & experimental systems

n/a	Involved in the study
☒	☐ Antibodies
☒	☐ Eukaryotic cell lines
☒	☐ Palaeontology and archaeology
☒	☐ Animals and other organisms
☐	☒ Clinical data
☒	☐ Dual use research of concern
☒	☐ Plants

Methods

n/a	Involved in the study
☒	☐ ChIP-seq
☒	☐ Flow cytometry
☒	☐ MRI-based neuroimaging

Clinical data

Policy information about clinical studies

All manuscripts should comply with the ICMJE guidelines for publication of clinical research and a completed CONSORT checklist must be included with all submissions.

Clinical trial registration	This study was registered at the Chinese Clinical Trial Registry, Registration number: ChiCTR2300073613.
Study protocol	An overview of this clinical trial can be accessed via this link: https://www.chictr.org.cn/showproj.html?proj=173173; for detailed protocol procedures, please refer to the Method section in this article.
Data collection	As mentioned in the recruitment section, the Discovery set from center 1 (the Second Affiliated Hospital of Shantou University Medical College) was collected between 2016 and 2020. Validation set from center 2 (Cancer Hospital of Shantou University Medical College) was obtained between 2021 and 2022. Test set from center 3 (Shantou Central Hospital) were collected between 2021 and 2022.
Outcomes	We pre-defined the primary outcome measures based on their clinical relevance and importance in evaluating the efficacy of our diagnostic approach. In this study, the primary outcomes include metabolite levels (measured through NMR and MS metabolomics) and indicators such as AUC (95% CI). Secondary outcome measures were also pre-defined to capture additional relevant information. The secondary outcome in this study refers to the levels of relevant metabolic enzymes (obtained through TCGA database analysis) and indicators such as sensitivity and specificity.
