# Supplementary material for: NMR and MS reveal characteristic metabolome atlas and optimize esophageal squamous cell carcinoma early detection
Source: Nat Commun. 2024 Mar 19;15:2463. doi: 10.1038/s41467-024-46837-0 (PMC10951220; doi:10.1038/s41467-024-46837-0)
Supplement: Supplementary file 4 — Description of Additional Supplementary Data Files [file 41467_2024_46837_MOESM4_ESM.docx]

**Description of Additional Supplementary Data Files**

File Name: Supplementary Data 1

Description: Key differential metabolites screening in ESCC tissues incorporating NMR and MS-based metabolomics data.

File Name: Supplementary Data 2

Description: Resonance assignments of metabolites in ^1^H-NMR spectra of tissues, serum and urine, with characteristic peaks underlined.

File Name: Supplementary Data 3

Description: Statistical analysis of the differential metabolites in tissues by NMR and MS, and in sera and urine by NMR.

File Name: Supplementary Data 4

Description: Metabolite contents of modules in the tissue-comparing and serum-comparing identified by WGCNA.
